# Interrelations of Justice, Rejection, Provocation, and Moral Disgust Sensitivity and Their Links with the Hostile Attribution Bias, Trait Anger, and Aggression

**DOI:** 10.3389/fpsyg.2016.00795

**Published:** 2016-05-30

**Authors:** Rebecca Bondü, Philipp Richter

**Affiliations:** ^1^Department of Psychology, University of KonstanzKonstanz, Germany; ^2^Department of Psychology, University of PotsdamPotsdam, Germany

**Keywords:** justice sensitivity, rejection sensitivity, provocation sensitivity, moral disgust sensitivity, trait anger, hostile attribution bias, aggression

## Abstract

Several personality dispositions with common features capturing sensitivities to negative social cues have recently been introduced into psychological research. To date, however, little is known about their interrelations, their conjoint effects on behavior, or their interplay with other risk factors. We asked *N* = 349 adults from Germany to rate their justice, rejection, moral disgust, and provocation sensitivity, hostile attribution bias, trait anger, and forms and functions of aggression. The sensitivity measures were mostly positively correlated; particularly those with an egoistic focus, such as victim justice, rejection, and provocation sensitivity, hostile attributions and trait anger as well as those with an altruistic focus, such as observer justice, perpetrator justice, and moral disgust sensitivity. The sensitivity measures had independent and differential effects on forms and functions of aggression when considered simultaneously and when controlling for hostile attributions and anger. They could not be integrated into a single factor of interpersonal sensitivity or reduced to other well-known risk factors for aggression. The sensitivity measures, therefore, require consideration in predicting and preventing aggression.

## Introduction

Recently, several individual difference measures have been introduced into psychological research that capture sensitivities to negative social cues, such as injustice, rejection, moral norm violations, or provocation, and shape perceptions of and emotional, cognitive, and behavioral responses toward these cues. Little, however, is known about how these measures interrelate, whether they can be considered discrete constructs, whether they can be distinguished from established similar trait measures, and how they work together in predicting behavior.

Justice sensitivity predisposes to frequent perceptions of and intense emotional and cognitive responses toward injustice from different perspectives, namely those of a victim, an observer, or a perpetrator (Schmitt et al., 1995). Rejection sensitivity predisposes to expecting, perceiving, and overreacting to potential rejection (Downey and Feldman, 1996). Provocation sensitivity predisposes to perceiving others’ behavior as provoking (Lawrence, 2006). Moral disgust sensitivity predisposes to feelings of disgust in the face of moral norm violations (Tybur et al., 2009; **Table 1**). Hence, all sensitivities constitute social-cognitive trait measures that may influence and explain interpersonal differences in response to situational factors (Mischel and Shoda, 1995). Despite potential similarities, coming from different fields of psychology, these measures have rarely been considered simultaneously. Thus, little or nothing is known about their interrelations and similarities and whether they can be considered discrete constructs. The first goal of the present study, therefore, was to investigate whether justice, rejection, moral disgust, and provocation sensitivity may be integrated into one single factor of interpersonal sensitivity (e.g., unjust treatment, social rejection, and moral norm violations could all be interpreted as forms of provocation) or should be considered discrete constructs, in order to add to a better understanding of these measures.

**Table 1:** Definitions of and overview over the sensitivity measures in the present study.

Justice Sensitivity	The tendency to perceive and intensely respond to injustice… … to one’s own disadvantage → victim sensitivity … to the disadvantage of others, caused by others → observer sensitivity … to the disadvantage of others, caused by oneself → perpetrator sensitivity (Schmitt et al., 1995, 2010)
Rejection Sensitivity	The tendency to expect, readily perceive, and overreact to rejection (Downey and Feldman, 1996, p. 1327).
Provocation Sensitivity	The tendency to perceive others’ behavior as provoking (Lawrence, 2006)
Moral Disgust (Sensitivity)	The tendency to experience disgust at moral norm violations (e.g., Tybur et al., 2009)

The sensitivity measures are not only theoretically similar, but also have similar effects on behavior. For example, all have been linked to aggression: Higher victim justice, rejection, and provocation sensitivity predicted higher aggression (Downey et al., 1998; Lawrence, 2006; Ayduk et al., 2008; Bondü and Krahé, 2015); higher observer and perpetrator justice and moral disgust sensitivity predicted lower aggression (Pond et al., 2011; Bondü and Krahé, 2015). Aggression, therefore, is a valuable outcome measure in order to examine similarities between the sensitivity measures as well as their interplay in and their independent contributions to predicting behavior. Furthermore, it is important to identify variables that may promote aggression in order to develop effective prevention measures. The second aim of our study, therefore, was to investigate the conjoint contribution of the sensitivity measures to the prediction of aggression in order to examine the interplay of the sensitivity measures in predicting behavior and to identify the most influential potential risk factors for aggression among these measures.

Finally, the sensitivity measures evoke emotional and cognitive responses to the relevant cues that resemble other important and well-established emotional and cognitive trait risk factors for aggression, that is, trait anger and the hostile attribution bias. Victim, rejection, moral disgust, and provocation sensitivity are related to anger; observer sensitivity is linked to indignation, a moral emotion similar to anger (Schmitt et al., 1995; Lawrence, 2006). Trait anger reliably predicts aggression, particularly reactive aggression that is shown in response to perceived provocation (Crane and Testa, 2014). Furthermore, victim and rejection sensitivity have been compared to the hostile attribution bias (Gollwitzer et al., 2013; Bondü and Esser, 2015). The hostile attribution bias is defined as the tendency to ascribe negative, hostile intent to others’ ambiguous behavior (Epps and Kendall, 1995). Similarly, people high in disgust sensitivity are prone to ascribing evil intent to others (Jones and Fitness, 2008). Thus, some sensitivity measures show overlaps with trait anger and hostile attributions. Hence, the third aim of our study was to investigate whether the sensitivity measures add to the prediction of aggression above and beyond the hostile attribution bias and trait anger as well-established affective and cognitive risk factors for aggression or whether their effects can be explained by these factors. If this were not the case, at least some of the sensitivity measures would require stronger consideration as risk factors for aggression by future research and when developing prevention and intervention measures against aggression.

Thus, the present study seeks to add to a better understanding of the sensitivity measures, their links to other risk factors for, and their effects on aggression. It connects research questions from personality and social psychology and adds to the ongoing conversation about the meaning of contextual or social-cognitive personality variables (sensitivity measures) as compared to more global personality variables (e.g., trait anger).

### Aggressive Behavior

According to a frequently used definition, aggression encompasses behavior that aims at harming another person who strives to avoid this harm (Baron and Richardson, 1994). Aggression is not a homogeneous construct. Mostly, physical (e.g., hitting), verbal (e.g., insulting), and relational (e.g., gossiping) *forms* of aggression are distinguished. Physical and verbal aggression is more frequent in males. Some studies found relational aggression to be more frequent among females than among males, but mostly gender differences seemed negligible (Archer, 2004; Card et al., 2008; Smith et al., 2009).

Furthermore, reactive and proactive *functions* of aggression are distinguished. Reactive aggression is defined as aggression in response to perceived negative treatment in order to defend or retaliate and also referred to as hostile aggression. Reactive aggression is related to anxiety, depression, or peer rejection. Proactive aggression is defined as unprovoked aggression that is utilized to attain egoistic goals (e.g., status, fun) and also referred to as instrumental aggression. Proactive aggression is associated with negative, but also with positive outcomes, such as higher peer status (Vitaro et al., 2006).

### Sensitivity Measures

The sensitivity measures in the present study (**Table 1**) share several features. First, all capture vulnerabilities to negative social cues, reflect interpersonal differences in response to situational stimuli (Lawrence, 2006), and form social-cognitive personality traits that guide individuals’ behavior in certain situations (e.g., Mischel and Shoda, 1995; Fleeson and Jayawickreme, 2015). Second, justice, rejection, and moral disgust sensitivity showed low to moderate correlations with neuroticism (Downey and Feldman, 1996; Schmitt et al., 2005; Chapman and Anderson, 2013), indicating overlaps with this broad personality dimension, but also large amounts of variance that cannot be explained by neuroticism. Third, all sensitivities should predispose to hypervigilance toward the respective relevant cues, expectations of their occurrence, their frequent and intense perceptions, and negative interpretations of ambiguous situations (e.g., Baumert et al., 2010). Fourth, all measures require a negative appraisal of others’ or one’s own behavior. Finally, all sensitivity measures shape the cognitive, emotional, and/or behavioral responses toward the respective cues.

#### Justice Sensitivity

Justice sensitivity predisposes to perceiving injustice from several perspectives. Justice-sensitive individuals are generally prone to rumination as the cognitive response toward injustice. Highly victim-sensitive individuals readily perceive themselves as victims of injustice and are prone to respond with anger and retaliation; highly observer-sensitive individuals easily perceive injustice to the disadvantage of others and are prone to indignation, victim compensation, and perpetrator punishment; highly perpetrator-sensitive individuals readily perceive themselves as causing injustice and are prone to guilt, victim compensation, and self-punishment (Schmitt et al., 2005, 2010).

Reflecting a conjoint concern for justice, all justice-sensitivity perspectives are positively correlated. Victim sensitivity, however, also comprises egoistic concerns for justice and correlates with jealousy (Schmitt et al., 2005), hostility (Schmitt et al., 2010), and antisocial behavior (Fetchenhauer and Huang, 2004; Gollwitzer et al., 2005; Bondü and Krahé, 2015). Victim-sensitive individuals are thought to have a suspicious mindset, to attribute hostile intent, and to readily perceive cues of untrustworthiness, causing them to withdraw cooperation in order to avoid being taken advantage of in the alleged presence of these cues (Gollwitzer et al., 2013). Thus, similar to the hostile attribution bias, victim sensitivity may create expectations of others’ negative behavior similar to dysfunctional thoughts (Bondü and Esser, 2015). In children and adolescents, there were no gender differences in victim sensitivity or its relations with aggression (Bondü and Krahé, 2015). In adults, men reported higher victim sensitivity (Schmitt et al., 2010), but links with aggression were more pronounced in women (Bondü and Richter, 2015).

Observer and perpetrator justice sensitivity reflect altruistic concerns for justice and positively correlate with empathy (Schmitt et al., 2005) as well as prosocial behavior (Fetchenhauer and Huang, 2004; Gollwitzer et al., 2005) and negatively correlate with antisocial behavior (Gollwitzer et al., 2005, 2013; Bondü and Esser, 2015; Bondü and Krahé, 2015). Evidence for prosocial effects of perpetrator sensitivity is unequivocal, but observer sensitivity may apparently promote reactive aggression (Lotz et al., 2011; Bondü and Krahé, 2015). Females reported higher observer and perpetrator sensitivity than males (Schmitt et al., 2010; Bondü and Elsner, 2015), but there were no gender differences in the links with aggression among boys and girls (Bondü and Krahé, 2015).

#### Rejection Sensitivity

In adults, rejection sensitivity is defined as the concern and expectation of not being accepted (Downey and Feldman, 1996). It is related to a range of mental health problems, including depressive, anxiety, or borderline symptoms (Rosenbach and Renneberg, 2011). Regarding aggression, rejection-sensitive adults are prone to jealousy, hostility, and aggression in partner relationships (Downey et al., 2000; Romero-Canyas et al., 2010). Furthermore, rejection sensitivity moderated the link between rejection in a computer game and aggression (Ayduk et al., 2008). Rejection sensitivity has been assumed to work similarly to the hostile attribution bias as well (Bondü and Krahé, 2015). Links between rejection sensitivity and aggression were similar for boys and girls (Bondü and Krahé, 2015).

#### Provocation Sensitivity

So far, little is known about the affective and cognitive processes underlying provocation sensitivity. In line with theoretical assumptions, however, highly provocation-sensitive adults tended to perceive others’ behavior as provoking (Lawrence and Hodgkins, 2009). Provocation sensitivity was associated with physical aggression (Lawrence, 2006) and predicted aggression after provocation (Lawrence and Hutchinson, 2012), thus backing the assumption that provocation is an important cause of aggression (Anderson and Bushman, 2001). Those high and low in provocation sensitivity did not differ in hostility and trait anger (Lawrence, 2006). There were no gender differences in provocation sensitivity, but particularly women reported provocation to elicit aggression (Lawrence, 2006).

#### Moral Disgust Sensitivity

Not only physically aversive stimuli (e.g., feces) may evoke disgust, but also moral norm transgressions (Chapman and Anderson, 2013). Individuals differ in their tendency to feel disgust at moral norm violations (Tybur et al., 2009). General (primarily physical) disgust sensitivity and disease avoidance is related to conservative values, authoritarianism, the advocation of social exclusion of norm violators, and the attribution of evil intent (Faulkner et al., 2004; Jones and Fitness, 2008). These finding suggest positive correlations between disgust sensitivity and aggression and, therefore, some have argued that disgust sensitivity may be driven by anger rather than reflecting disgust in the narrow sense (Simpson et al., 2006). However, if disgust genuinely served as a moral emotion in moral disgust sensitivity and because disgust is a withdrawal-orientated emotion, (moral) disgust as opposed to anger should inhibit aggression. In line with this reasoning, Pond et al. (2011) found moral disgust to predict less physical, verbal, and reactive aggression and less approval of intimate partner violence as well as to not relate to hostility in experimental studies. Women show higher moral disgust than men (Tybur et al., 2009). The moderating role of gender in the link of disgust sensitivity and aggression is not yet well understood.

#### Similarities and Links between the Sensitivity Measures

Given the similarities, we expected all sensitivity measures to positively correlate. These correlations should be particularly pronounced between measures with an egoistic focus, that is, victim, rejection, and provocation sensitivity, and measures with a moral/altruistic focus, that is, observer, perpetrator, and moral disgust sensitivity. According to previous research, justice and rejection sensitivity cannot be integrated into a single factor of interpersonal sensitivity (Bondü and Esser, 2015; Bondü and Richter, 2015). Thus, we also expected the other sensitivities to form discrete measures that independently add to the prediction of aggression. Victim, rejection, and provocation sensitivity should predict higher aggression, particularly reactive aggression, which is triggered by alleged negative social interactions. Observer, perpetrator, and moral disgust sensitivity should predict less aggression, particularly proactive aggression, because moral concerns associated with these sensitivities should prevent from using aggression to reach egoistic goals.

### Other Risk Factors for Aggression

#### Hostile Attribution Bias

The tendency to attribute hostile intent in order to explain others’ ambiguous behavior (Epps and Kendall, 1995) has been linked to aggression—particularly reactive aggression—in different age groups (Dodge and Crick, 1990; Epps and Kendall, 1995; Bailey and Ostrov, 2007). It has been outlined as one key risk factor for aggressive behavior in the social information processing model (Crick and Dodge, 1996) and by subsequent research (Orobio de Castro et al., 2002), indicating that people with a strong hostile attribution bias in particular tend to respond aggressively to perceived provocation. There were no gender differences in hostile attributions in children (Cillessen et al., 2014) or emerging adults (Bailey and Ostrov, 2007), but boys revealed stronger links between hostile attributions and aggression than girls (Cillessen et al., 2014).

#### Trait Anger

Anger as an approach-oriented emotion that prepares for and enables a fight response has been linked to aggressive behavior, particularly to reactive aggression, in different age groups (Hubbard et al., 2010; Crane and Testa, 2014). Trait anger predisposes to frequent and intense anger in various situations (Buss and Perry, 1992) and, consequently, to reactive aggression in particular. In a representative German sample, there were no gender differences in trait anger (Rohrmann et al., 2013).

#### Potential Links to Sensitivity Measures

We expected the egoistic-focused sensitivity measures to positively relate to hostile attributions and anger. Reflecting moral/altruistic concerns, we expected observer and perpetrator sensitivity to negatively relate to hostile attributions and trait anger. Because disgust has been linked to the attribution of evil intentions (Jones and Fitness, 2008), we expected positive links with hostile attributions. However, we assumed that the emotion underlying moral disgust sensitivity is disgust and, therefore, expected small—if any—correlations with trait anger.

### The Present Study

The present study examined the links of justice, rejection, moral disgust, and provocation sensitivity, their associations with the hostile attribution bias and trait anger, and their interplay in predicting forms and functions of aggression in an adult sample using structural equation models. Our study adds to the understanding of individual difference variables pertaining to sensitivities to social cues and their relations to negative social behavior as well as potentially related broader trait measures. Based on theoretical considerations and previous research results, we derived the following hypotheses:

Hypothesis 1: Victim, observer, perpetrator, rejection, provocation, and moral disgust sensitivity form positively related, but discrete measures rather than combining into a single factor of interpersonal sensitivity.
Hypothesis 2: (a) The sensitivity measures can be separated from the hostile attribution bias and trait anger. (b) Victim, rejection, and provocation sensitivity are positively related to the hostile attribution bias and trait anger. (c) Observer and perpetrator sensitivity are negatively related to the hostile attribution bias and trait anger. (d) Moral disgust sensitivity is positively related to hostile attributions only.
Hypothesis 3: Victim, rejection, and provocation sensitivity positively predict aggression; perpetrator and moral disgust sensitivity negatively predict aggression.
Hypothesis 4: These effects hold stable when the hostile attribution bias and trait anger are entered into the model.

We also examined potential gender differences in prediction patterns.

## Materials and Methods

### Sample

The sample included *N* = 349 German adults (70.8% female). Participants were recruited via E-mail, social networks, and notices in university buildings. Age ranged between 18 and 75 years (*M* = 27.2, *SD* = 10.37); 242 participants (69.3%) were students (78.5% female), 17 (4.9%) visited school or were in vocational training, 62 (20.6%) worked as salaried personnel or freelancers, 6 (1.7%) were unemployed, and 12 (3.4%) had another occupational status. Given the high percentage of students and participants in vocational training, the average income per month was low: 18.7% participants had no own income, 55.2% an income below 1000 euro, 20.2% an income between 1000 and 1999 euro, and 5.7% an income of 2000 euro and above.

### Measures

**Table 2** shows the internal consistencies of all dependent and independent measures in the present study.

**Table 2:** Descriptive statistics and gender differences.

Scale	α	Total *M* (*SD*)	Male *M* (*SD*)	Female *M* (*SD*)
Justice sensitivity–Victim Justice sensitivity–Observer∗ Justice sensitivity–Perpetrator∗ Rejection sensitivity Moral Disgust Provocation Sensitivity Hostile attributions∗ Anger Physical aggression∗∗∗ Verbal aggression∗∗ Relational aggression Proactive aggression Reactive aggression	0.78 0.81 0.82 0.68 0.81 0.86 0.70 0.83 0.78 0.68 0.79 0.87 0.91	3.31 (0.97) 3.38 (0.87) 3.69 (0.95) 8.21 (3.42) 5.32 (0.68) 3.16 (0.67) 1.62 (0.33) 2.42 (0.57) 1.85 (0.63) 2.61 (0.54) 1.57 (0.67) 1.60 (0.60) 2.76 (0.87)	3.15 (0.95) 3.20 (0.95) 3.50 (1.01) 7.96 (3.41) 5.39 (0.74) 3.12 (0.66) 1.59 (0.32) 2.43 (0.57) 2.23 (0.70) 2.73 (0.61) 1.61 (0.72) 1.63 (0.66) 2.66 (0.85)	3.40 (0.96) 3.43 (0.83) 3.76 (0.92) 8.35 (3.45) 5.30 (0.66) 3.18 (0.68) 1.64 (0.33) 2.41 (0.57) 1.71 (0.53) 2.57 (0.51) 1.57 (0.66) 1.59 (0.57) 2.80 (0.87)

*Significant gender differences: ∗*p* < 0.05, ^∗∗^*p* < 0.01, ^∗∗∗^*p* < 0.001; *N* = 339 (*n* men = 100, *n* women = 239).*

#### Justice Sensitivity

We measured victim (“I cannot easily bear it when others take advantage of me”), observer (“I cannot easily bear it when someone takes advantage of others”), and perpetrator justice sensitivity (“I cannot easily bear the feeling of taking advantage of someone”; 0 *strongly disagree* to 5 *strongly agree*, respectively) with the 5-items-per-scale version of the Justice Sensitivity Inventory (Bondü and Elsner, 2015). We computed mean values for each perspective. Evidence for the reliability and validity of the original and the 5-item measure has been provided (Schmitt et al., 2005, 2010; Bondü and Elsner, 2015).

#### Rejection Sensitivity

We measured rejection sensitivity with a translated version of the Adult Rejection Sensitivity Questionnaire (Berenson et al., 2009). Participants were presented with nine situations possibly resulting in rejection (“You ask your parents for extra money to cover living expenses”) and rated how anxious they would feel about rejection (1 *very unconcerned* to 6 *very anxious*) as well as the likelihood of rejection (1 *very unlikely* to 6 *very likely*). Mean rejection sensitivity was computed by multiplying the anxiety ratings with the reversed likelihood-of-rejection ratings per situation and dividing their sum by nine (Berenson et al., 2009). Evidence for the reliability and validity of the original questionnaire has been provided (Berenson et al., 2009).

#### Provocation Sensitivity

We measured provocation sensitivity with 12 translated items (“I feel aggressive when someone insults me”) from the Situational Triggers of Aggressive Responses scale (Lawrence, 2006). Response options ranged from (1) *totally disagree* to (5) *totally agree*. We computed mean values. The original measure has been shown to be reliable and valid (Lawrence, 2006).

#### Moral Disgust Sensitivity

We measured moral disgust sensitivity using four translated items from the Three Domains of Disgust Scale (“Forging someone’s signature on a legal document”; Tybur et al., 2009) and 12 translated items from Hutcherson and Gross (2011; “A company executive refuses to sit next to a laborer on a train”). Response options ranged from (1) *not at all disgusting* to (7) *extremely disgusting*. We computed mean values. Both original measures have been reported to be reliable and valid (Tybur et al., 2009; Hutcherson and Gross, 2011).

#### Hostile Attribution Bias

We measured attributions of hostile intent with four translated scenarios from the Social Information Processing–Attribution Bias Questionnaire (Coccaro et al., 2009). Each scenario described a situation with a negative outcome that may have been caused intentionally or unintentionally. Participants rated the likelihood of two explanations for the outcome signaling hostile intent (“My co-worker wanted to burn me with hot coffee”) per scenario from (1) *very unlikely* to (4) *very likely.* We computed mean hostile attribution bias scores across the eight items. The original measure has been shown to reliably and validly measure the hostile attribution bias (Coccaro et al., 2009).

#### Trait Anger

We measured trait anger with seven items (“I sometimes fell like a powder keg ready to explode”) from the translated version of the Aggression Questionnaire (Buss and Perry, 1992; Krahé and Möller, 2010). Response options ranged from (1) *totally disagree* to (5) *totally agree*. We computed mean values (see below for information on reliability and validity).

#### Forms and Functions of Aggression

We measured physical (“Given enough provocation, I may hit another person,” nine items), verbal (“I can’t help getting into arguments when people disagree with me,” five items), and relational aggression (“When it serves my interests, I sometimes play people off against each other,” four items), with a translated version of the Aggression Questionnaire (Buss and Perry, 1992; Buss and Warren, 2000; Krahé and Möller, 2010). Response options ranged from (1) *totally disagree* to (5) *totally agree.* We computed mean values for each scale. The Aggression Questionnaire has been shown to be a reliable and valid measure (e.g., Aitken Harris, 1997; Buss and Warren, 2000).

Participants rated functions of aggression with three items per form of aggression they had reported to show, using an adapted version of the Instrument for Reactive and Proactive Aggression (Polman et al., 2009). Response options ranged from (1) *never* to (5) *very often*. By averaging the function ratings across forms, total scores for reactive (“Because you felt pressured or harassed”) and proactive (“To demonstrate your superiority”) aggression were calculated. We excluded 10 participants who did not report any forms of aggression from the analyses on functions of aggression, because participants who did not show any aggression also cannot name any reasons for showing this behavior. Polman et al. (2009) provided evidence for the reliability and validity of the original measure.

### Procedure

We collected the data via an online survey between September and December 2013. All participants attended voluntarily, were guaranteed privacy, and given the chance to win 1 out of 10 vouchers for an online retail company. In addition to the competition, university students received course credit for their participation. The survey was programmed to force answers. Due to program mistakes, however, there were isolated missing values on single variables. Due to the low percentage of missing values we used single imputation to replace them.

## Results

### Descriptive Statistics and Confirmatory Factor Analyses

**Table 2** shows internal consistencies, mean values, and standard deviations of all measures for the total sample and separately for men and women. Gender differences were examined via a MANCOVA controlling for age. There was a significant multivariate main effect of gender: *F*(13,324) = 7.786 (*p* < 0.001), $\eta_{p}^{2}$ = 0.238. Women reported significantly higher observer sensitivity (*p* = 0.028), perpetrator sensitivity (*p* = 0.021), and hostile attributions (*p* = 0.045). Men reported significantly higher physical (*p* < 0.001) and verbal (*p* = 0.005) aggression.

Age was negatively related to victim and rejection sensitivity as well as proactive and relational aggression and positively related to moral disgust sensitivity as well as hostile attributions. Mostly in line with Hypothesis 1, we found positive correlations between all sensitivity measures except for null-correlations of rejection sensitivity with the justice sensitivity measures and moral disgust as well as of victim and moral disgust sensitivity. In line with Hypothesis 2b, victim and provocation sensitivity were positively correlated with hostile attributions and trait anger. Contrasting Hypothesis 2b, rejection sensitivity was only related to trait anger. Contrasting Hypothesis 2c, observer and perpetrator sensitivity were unrelated to hostile attributions and trait anger. In line with Hypothesis 2d, disgust sensitivity showed a positive correlation with hostile attributions, but no correlation with trait anger.

Regarding aggression, provocation sensitivity, hostile attributions, and trait anger showed the expected positive correlations with all aggression measures. Victim and rejection sensitivity were positively correlated with most aggression measures, but were uncorrelated to two out of five of these measures, respectively. Perpetrator and moral disgust sensitivity showed the expected negative correlations with the aggression measures with few exceptions (i.e., both were unrelated to reactive aggression and moral disgust was unrelated to physical aggression). Observer sensitivity was unrelated to most aggression measures (**Table 3**).

**Table 3:** Zero-order correlations of sensitivity measures, hostility, anger, aggression measures, and age in the total sample.

		2	3	4	5	6	7	8	9	10	11	12	13	14
1	JS-Victim	0.36^∗∗∗^	0.22^∗∗∗^	0.10	0.04	0.34^∗∗∗^	0.17^∗∗∗^	0.26^∗∗∗^	0.06	0.10	0.15^∗∗^	0.12∗	0.29^∗∗∗^	-0.25^∗∗∗^
2	JS-Observer	-	0.57^∗∗∗^	-0.04	0.20^∗∗∗^	0.15^∗∗^	0.03	0.07	0.05	0.06	-0.01	-0.01	0.05	-0.02
3	JS-Perpetrator		-	0.01	0.29^∗∗∗^	0.16^∗∗^	-0.04	-0.03	-0.11∗	-0.12∗	-0.14∗	-0.20^∗∗∗^	0.03	-0.01
4	Rejection sensitivity			-	-0.07	0.16^∗∗^	0.09	0.21^∗∗∗^	0.17^∗∗^	-0.06	0.12∗	-0.05	0.25^∗∗∗^	-0.13∗
5	Moral Disgust				-	0.20^∗∗∗^	0.20^∗∗∗^	-0.01	-0.07	-0.14^∗∗^	-0.15^∗∗^	-0.20^∗∗∗^	-0.05	0.33^∗∗∗^
6	Provocation sensitivity.					-	0.24^∗∗∗^	0.38^∗∗∗^	0.24^∗∗∗^	0.20^∗∗∗^	0.16^∗∗^	0.14∗	0.42^∗∗∗^	0.02
7	Hostile attribute						-	0.23^∗∗∗^	0.10	0.15^∗∗^	0.14∗	0.16^∗∗^	0.18^∗∗∗^	0.15^∗∗^
8	Trait Anger							-	0.36^∗∗∗^	0.39^∗∗∗^	0.29^∗∗∗^	0.22^∗∗∗^	0.39^∗∗∗^	-0.03
9	Physical aggression								-	0.29^∗∗∗^	0.35^∗∗∗^	0.19^∗∗∗^	0.36^∗∗∗^	0.05
10	Verbal aggression									-	0.28^∗∗∗^	0.37^∗∗∗^	0.27^∗∗∗^	-0.05
11	Relational aggression										-	0.31^∗∗∗^	0.34^∗∗∗^	-0.16^∗∗^
12	Reactive aggression											-	0.26^∗∗∗^	-0.00
13	Proactive aggression												-	-0.20^∗∗∗^
14	Age													-

*^∗^*p* < 0.05; ^∗∗^*p* < 0.01; ^∗∗∗^*p* < 0.001.*

**FIGURE 1:**
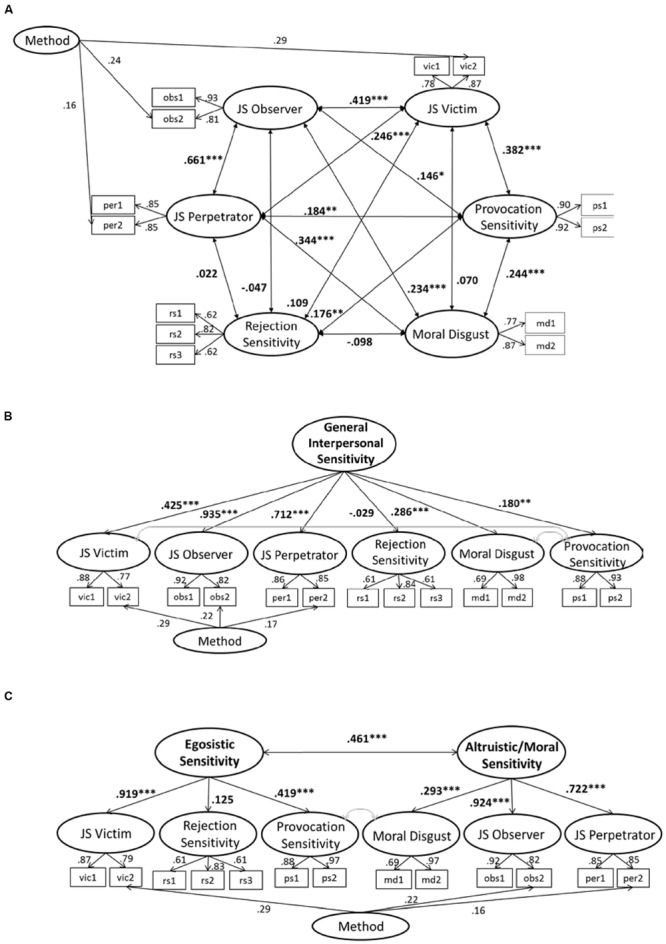
**Comparison of three different Confirmatory Factor Analysis (CFA) models.** Results of the χ^2^-difference test and comparisons of absolute fit indices indicate that model a with six discrete, but correlated sensitivity factors shows the best fit with the data. **(A)** CFA model with discrete, interrelated sensitivity factors: χ^2^ = 91.985, *df* = 48, *p* < 0.001, RMSEA = 0.051, CFI = 0.975, SRMR = 0.030, *N* = 349. **(B)** CFA model with a second order factor of general interpersonal sensitivity: χ^2^ = 117.677, *df* = 55, *p* < 0.001, RMSEA = 0.057, CFI = 0.964, SRMR = 0.054, *N* = 349. **(C)** CFA model with two second order factors reflecting primary egoistic and altruistic/moral concerns: χ^2^ = 115.042, *df* = 51, *p* < 0.001, RMSEA = 0.057, CFI = 0.966, SRMR = 0.048, *N* = 349.

To test whether the six sensitivity measures can be sufficiently separated from another, we conducted a Confirmatory Factor Analysis (CFA). Latent factors were indicated by test-halves, rejection sensitivity was indicated by test-thirds (see below). An MLM estimator accounted for non-normally distributed data. The model with six discrete, but correlated latent sensitivity factors showed a good fit with the data [χ^2^(*df* = 48) = 91.985, *p* < 0.001, RMSEA = 0.051, CFI = 0.975, SRMR = 0.030, *N* = 349] and a significantly better fit than a model with a second order factor of general interpersonal sensitivity [even if correlations of error terms of provocation sensitivity with victim and moral disgust sensitivity were allowed and estimated; χ^2^(*df* = 55) = 117.677, *p* < 0.001, RMSEA = 0.057, CFI = 0.964, SRMR = 0.054, *N* = 349; Δχ^2^= 25.692, Δ*df* = 7, Δ*p* < 0.001]. It also fit the data better than a model with two correlated second order factors reflecting an egoistic and altruistic/moral focus [even if correlations of error terms of provocation and moral disgust sensitivity were allowed and estimated; χ^2^(*df* = 51) = 115.042, *p* < 0.001, RMSEA = 0.056, CFI = 0.966, SRMR = 0.048, *N* = 349; Δχ^2^= 23.057, Δ*df* = 7, Δ*p* = 0.002]. Supporting Hypothesis 1, this indicates that the sensitivities form discrete measures rather than combining into a single factor of general interpersonal sensitivity or into two factors with egoistic and altruistic focus (**Figure 1**). Given the low factor loadings of the rejection sensitivity measure in these models, we repeated the analyses excluding rejection sensitivity. The pattern of results did not change and also indicated a better fit of the model including five correlated, but discrete sensitivity factors than the models including second order factors. Hence, rejection sensitivity alone did not explain for the differences between measures and also the five other sensitivity measures should be considered discrete measures.

Finally, a further CFA including all six distinct sensitivity measures, hostile attributions, and trait anger and allowing all factors to correlate, also showed a good fit with the data [χ^2^(*df* = 89) = 132.666, *p* = 0.002, RMSEA = 0.037, CFI = 0.980, SRMR = 0.031, *N* = 349]. This indicates that in line with Hypothesis 2a, the sensitivity measures can be separated from hostile attributions and trait anger as well.

### Linking Sensitivity Measures, Hostile Attributions, and Trait Anger to Aggression

To examine the joint effects of the sensitivity measures, hostile attributions, and trait anger on forms and functions of aggression, we specified structural equation models using *Mplus*7 (Muthén and Muthén, 1998–2012). Latent factors were indicated by test-halves except for rejection sensitivity which was indicated by test-thirds (initial CFAs of the rejection sensitivity measure indicated a substantially better fit with the data if it was indicated by test-thirds instead of test-halves). A methods factor with loadings of all second test-halves from the justice-sensitivity subscales accounted for variance due to similar item wordings of the justice-sensitivity subscales (displayed as “methods factor” in the figures). All indicators showed significant loadings on their latent variables. We used an MLM-estimator to account for non-normally distributed data and conducted separate analysis for forms and functions of aggression controlling for age and gender. A CFA including all dependent and independent measures and with correlations between factors allowed and estimated confirmed the intended factor structure of distinct but interrelated factors [χ^2^(*df* = 297) = 441.942, *p* = 0.000, RMSEA = 0.038, CFI = 0.961, SRMR = 0.041, *N* = 339].

#### Forms of Aggression

**FIGURE 2:**
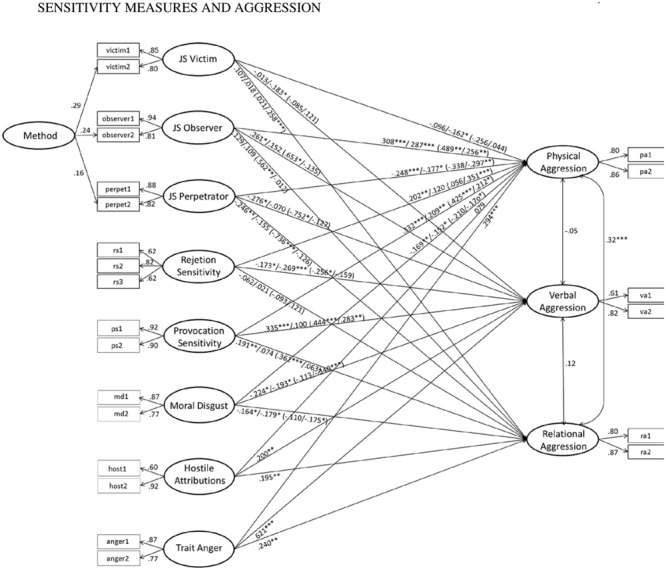
**Prediction of forms of aggression (total sample: controlled for gender and age; grouping model: controlled for age; correlations between predictors allowed and estimated).** Figures in front of slashes: path weights for the model only including the sensitivity measures, figures after slashes: path weights for the model also including hostile attributions and trait anger. Figures in brackets: path weights for men and women respectively. Total sample (only including sensitivity measures): χ^2^ = 230.816, *df* = 132, *p* < 0.001, RMSEA = 0.046, CFI = 0.962, SRMR = 0.039, *N* = 349; Total sample (also including hostile attribution bias and trait anger): χ^2^ = 303.183, *df* = 195, *p* < 0.001, RMSEA = 0.040, CFI = 0.966, SRMR = 0.038, *N* = 349; Grouping by gender (only including sensitivity measures): χ^2^ = 422.780, *df* = 266, *p* < 0.001, RMSEA = 0.058, CFI = 0.940, SRMR = 0.057, *N* = 349.

The path model for forms of aggression including only the sensitivity measures explained 40.7% variance in physical, 16.8% in relational, and 24.0% in verbal aggression (χ^2^= 230.816, *df* = 132, *p* < 0.001, RMSEA = 0.046, CFI = 0.962, SRMR = 0.039, *N* = 349). Mostly in line with Hypothesis 3, higher observer, rejection, and provocation sensitivity and lower perpetrator and moral disgust sensitivity predicted higher physical aggression. Higher observer and provocation sensitivity and lower perpetrator, rejection, and moral disgust sensitivity predicted higher verbal aggression. Higher provocation sensitivity and lower perpetrator and moral disgust sensitivity predicted higher relational aggression. Victim sensitivity did not add to the predictions (**Figure 2**).

When hostile attributions and trait anger were included in the model, higher trait anger predicted all three forms of aggression and higher hostile attributions predicted verbal and relational aggression; some of the previously significant effects of the sensitivity measures were non-significant (**Figure 2**; χ^2^= 303.183, *df* = 195, *p* < 0.001, RMSEA = 0.040, CFI = 0.966, SRMR = 0.038, *N* = 349). The model added to the amount of explained variance, explaining 47.4% variance in physical, 24.2% in relational, and 51.5% in verbal aggression. However, the model including only the sensitivity measures and the model also including hostile attributions and trait anger did not differ significantly according to χ^2^-difference test (Δχ^2^= 72.367, Δ*df* = 63, Δ*p* = 0.196). Also absolute fit indices indicated only small improvements of the model fit. Supporting Hypothesis 4, this indicates that the more parsimonious model explains the data equally well and should, therefore, be preferred.

Also when examining the moderating effect of gender, the more parsimonious multi-group model only including the sensitivity measures (χ^2^= 422.780, *df* = 266, *p* < 0.001, RMSEA = 0.058, CFI = 0.940, SRMR = 0.057, *N* = 349) was favored over the model also including hostile attributions and trait anger (χ^2^= 579.689, *df* = 392, *p* < 0.001, RMSEA = 0.052, CFI = 0.942, SRMR = 0.057, *N* = 349; Δχ^2^= 153.909, Δ*df* = 126, Δ*p* = 0.046; however, this model explained much larger amounts of variance, particularly in males, that is, 41.3% variance in physical, 42.2% in relational, and 73.7% in verbal aggression). In the multi-group model including only the sensitivity measures, factor loadings and intercepts were constrained to be equal, but path weights were allowed to vary between groups. The model revealed marked gender differences and fitted the data significantly better than a more constrained model with path weights constrained to be equal across groups (χ^2^= 494.905, *df* = 308, *p* < 0.001, RMSEA = 0.059, CFI = 0.929, SRMR = 0.080, *N* = 349; Δχ^2^= 72.125, Δ*df* = 42, Δ*p* = 0.003). It explained 29.7 and 26.9% variance in physical, 16.5 and 32.0% variance in relational, and 25.0 and 37.1% variance in verbal aggression in females and males, respectively. Physical aggression was predicted by higher observer and provocation sensitivity (marginally significant results for lower victim and perpetrator sensitivity, *p* = 0.057 and 0.063, respectively) in men and by higher observer, rejection, and provocation as well as by lower perpetrator and moral disgust sensitivity in women. Verbal aggression was predicted by higher observer and provocation as well as by lower perpetrator and rejection sensitivity in men and by higher provocation as well as lower moral disgust sensitivity in women. Relational aggression was predicted by higher observer and provocation as well as by lower perpetrator sensitivity in men and by lower moral disgust sensitivity in women.

#### Functions of Aggression

**FIGURE 3:**
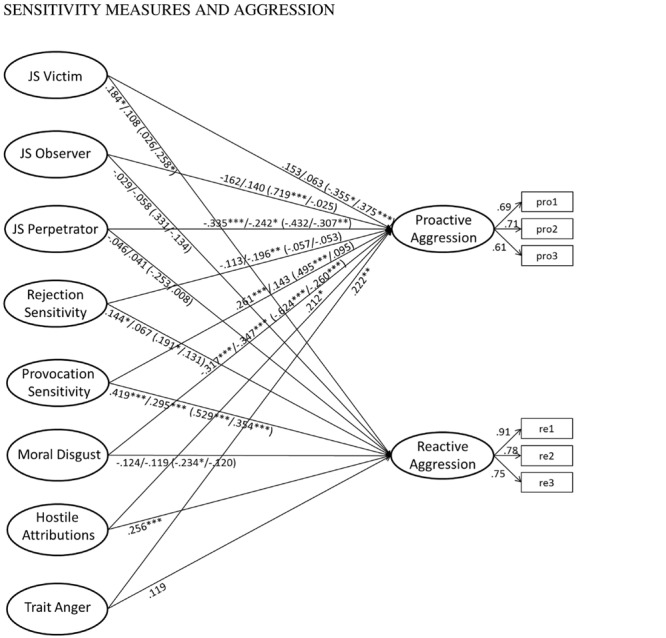
**Prediction of functions of aggression (total sample: controlled for gender and age; grouping model: controlled for age; correlations between predictors allowed and estimated; measurement model including method factor as in **Figure 1**).** Figures in front of slashes: path coefficients for the model only including the sensitivity measures; figures after slashes: path weights for the model also including hostile attributions and trait anger. Figures in brackets: path weights for men and women respectively. Total sample (only including sensitivity measures): χ^2^ = 250.652, *df* = 143, *p* < 0.001, RMSEA = 0.047, CFI = 0.958, SRMR = 0.044, *N* = 339; Total sample (also including hostile attribution bias and trait anger): χ^2^ = 310.093, *df* = 208, *p* < 0.001, RMSEA = 0.038, CFI = 0.967, SRMR = 0.041, *N* = 339; Grouping by gender (only including sensitivity measures): χ^2^ = 429.226, *df* = 286, *p* < 0.001, RMSEA = 0.054, CFI = 0.948, SRMR = 0.060, *N* = 339.

The model for functions of aggression including only the sensitivity measures (χ^2^= 250.652, *df* = 143, *p* < 0.001, RMSEA = 0.047, CFI = 0.958, SRMR = 0.044, *N* = 339) explained 25.5% variance in proactive and 32.7% in reactive aggression. Mostly in line with Hypothesis 3, provocation sensitivity positively and perpetrator and moral disgust sensitivity negatively predicted proactive aggression. Victim, rejection, and provocation sensitivity positively predicted reactive aggression (**Figure 3**).

When hostile attributions an trait anger were included in the model, higher trait anger predicted proactive and higher hostile attributions predicted proactive and reactive aggression; some of the previously significant effects of the sensitivity measures were non-significant (χ^2^= 310.093, *df* = 208, *p* < 0.001, RMSEA = 0.038, CFI = 0.967, SRMR = 0.041, *N* = 339). The model explained 32.6% variance in proactive and 38.7% in reactive aggression, but it did not differ from the more parsimonious model (Δχ^2^= 59.441, Δ*df* = 65, Δ*p* = 0.671), indicating that the model only including the sensitivity measures should be preferred. Supporting Hypothesis 4, absolute fit indices indicate a somewhat, but not substantially better model fit of the less parsimonious model as well.

Regarding the multi-group model for functions of aggression, the χ^2^-difference test indicated that the more parsimonious model only including the sensitivity measures (**Figure 3**; χ^2^= 429.226, *df* = 286, *p* < 0.001, RMSEA = 0.054, CFI = 0.948, SRMR = 0.060, *N* = 339) may be preferred over the model also including hostile attributions and trait anger (χ^2^= 558.693, *df* = 418, *p* < 0.001, RMSEA = 0.045, CFI = 0.956, SRMR = 0.053, *N* = 339; Δχ^2^= 129.467, Δ*df* = 132, Δ*p* = 0.546; however, again, the amount of explained variance of proactive aggression in men was substantially higher if hostile attributions and trait anger were included, i.e., 76%). The model revealed marked gender differences and showed a significant better fit with the data than a model with path weights constrained to be equal across groups (χ^2^= 496.196, *df* = 323, *p* < 0.001, RMSEA = 0.056, CFI = 0.935, SRMR = 0.081, *N* = 339; Δχ^2^= 66.970, Δ*df* = 37, Δ*p* = 0.002). It explained 32.4 and 54.3% variance in proactive and 31.7 and 43.2% variance in reactive aggression among women and men, respectively. Proactive aggression was predicted by higher observer and provocation as well as lower victim and moral disgust sensitivity in men (marginally significant effect of lower perpetrator sensitivity, *p* = 0.057) and by higher victim as well as lower perpetrator and moral disgust sensitivity in women. Reactive aggression was predicted by higher rejection and provocation as well as by lower moral disgust sensitivity in men and by higher victim and provocation sensitivity in women (marginally significant effect of higher rejection sensitivity, *p* = 0.062).

## Discussion

The present study examined the relations of justice, rejection, provocation, and moral disgust sensitivity, stable personality dispositions that capture vulnerabilities to distinct negative social cues and show theoretical and empirical overlaps. In line with our assumptions, however, only small to moderate positive correlations between the sensitivity measures and results from the CFA indicate that they cannot be integrated into a single factor of general interpersonal sensitivity, but should be treated and considered separately.

Because all sensitivity measures had been related to aggression previously and because preventing aggression is an important aim, we used aggression as an outcome measure in order to examine the interplay of the sensitivity measures in predicting behavior and to identify the most important potential risk factors for aggression among these measures. Different combinations of sensitivities predicted different forms and functions of aggression. Hence, simultaneously considering several sensitivities did not make any of them redundant. However, in no case all sensitivities added to the prediction of an aggression measure. In line with our assumptions, provocation sensitivity showed consistent positive associations with aggression, whereas perpetrator and moral disgust sensitivity showed quite consistent negative associations with aggression. Thus, these sensitivities seem to require special focus when researching aggression and developing prevention measures. Contrasting our expectations, observer sensitivity showed positive relations to aggression as well, particularly in men (cf. Bondü and Richter, 2015), whereas victim and rejection sensitivity revealed positive and negative relations with different aggression measures.

In line with our expectations, the sensitivity measures with an egoistic focus were mostly positively correlated to hostile attributions and trait anger, whereas moral disgust only related to hostile attributions. Correlations, however, were only small to moderate and CFA results indicated that the more specific sensitivity measures may be separated from the two broader trait measures. Considering hostile attributions and trait anger substantially added to the amount of explained variance in the aggression measures and previously significant effects of the sensitivity measures remained significant only in approximately one out of two cases. But χ^2^-difference tests and changes in absolute fit indices indicated that hostile attributions and trait anger did not improve the model fit significantly. Thus, they could not better explain the effects of the sensitivity measures on aggression. Hence, the sensitivities require consideration by future research on aggression and seem to have important impacts on behavior as would be postulated by the cognitive–affective system theory of personality (Mischel and Shoda, 1995).

### Differences and Similarities between the Sensitivity Measures

As outlined above, similarities between the sensitivity measures include a vulnerability and hypervigilance to negative social cues, negative evaluations of others’ and one’s own behavior, and intense negative affective, cognitive, and behavioral responses toward these cues. Thus, in line with our expectations, we mostly found positive correlations among the different sensitivity measures, irrespective of their egoistic or moral/altruistic focus. Correlations, however, were only small to moderate and CFA results indicated them to be distinct measures as well. Thus, despite theoretical and empirical overlaps, several differences between measures make them sufficiently distinguishable from one another. These differences include (**Table 4**):

**Table 4:** Overview over differences and similarities between sensitivity measures.

	Victim Justice Sensitivity	Observer Justice Sensitivity	Perpetrator Justice Sensitivity	Rejection Sensitivity	Provocation Sensitivity	Moral Disgust Sensitivity
Focus of Concern	Egoistic	Moral (egoistic?)	Moral	Egoistic	Egoistic	Moral (egoistic)
Affective/Cognitive Focus	Affective + cognitive	Affective + cognitive	Affective + cognitive	Affective + cognitive	Affective	Affective
Cognitive Response	Rumination + strain	Rumination + strain	Rumination + strain	Expectation of Rejection	-	-
Affective Response/Underlying Emotion	Anger	Indignation	Guilt	Anxiety (+Anger?)	Anger	Disgust
Time Perspective	Present	Present	Present	Future	Present	Present
Others’/Own Behavior	Unjust/immoral	Unjust/immoral	Unjust/immoral	Hurting	Provoking	Immoral
Relation Aggressive Behavior	Generally positive	Positive/negative	Negative	Positive/negative	Positive	Negative

#### Focus of Concern

Victim, rejection, and provocation sensitivity reflect egoistic concerns; observer, perpetrator, and moral disgust sensitivity reflect moral/altruistic concerns. Observers of injustice, however, may apparently focus on egoistic *or* altruistic concerns and identify with the victim or the perpetrator of injustice depending on the situation (Lotz et al., 2011; Bondü and Krahé, 2015) as indicated by positive and negative relations with the aggression measures (cf. Bondü and Richter, 2015). Similarly, moral concerns associated with moral disgust may not be altogether altruistic, but may also subsume egoistic interests in the long-term adherence of norms (Jones and Fitness, 2008) or the confirmation of a positive group status as described in the literature on altruistic punishment (e.g., Lotz et al., 2011). In addition, although all sensitivities in the present study require the appraisal of behavior as wrong or immoral, only perpetrator sensitivity refers to one’s own behavior. Hence, perpetrator sensitivity should have the strongest genuinely moral focus among these measures.

#### Affective/Cognitive Focus

As outlined above, all sensitivities require a cognitive–affective appraisal of behavior as (morally) wrong. However, only justice and rejection sensitivity questionnaires capture both affective *and* cognitive responses to unjust situations or situations that may result in rejection. In contrast, moral disgust and provocation sensitivity merely capture the intensity of affective responses to morally wrong or potentially provoking behavior. Given that moral disgust and provocation sensitivity tended to show the most consistent and strongest (positive and negative) relations with the aggression measures, this may indicate that the affective component of the sensitivity measures in particular explains the links of the sensitivity measures with the aggression measures.

#### Cognitive Responses

Justice sensitivity captures rumination as the central cognitive response to injustice and also considers the strain associated with these experiences (Baumert et al., 2014); rejection sensitivity captures the expectation of rejection as the primary cognitive response to rejection. Both cognitive responses may foster social withdrawal in order to avoid future victimization. They may, therefore, explain why all justice sensitivity subscales and rejection sensitivity also showed some negative relations with aggression as an approach-oriented behavior and why they were related to internalizing problems by previous research as well (Rosenbach and Renneberg, 2011; Bondü and Elsner, 2015).

#### Affective Responses

The central affect underlying victim and provocation sensitivity is anger. Indignation—the core emotion accompanying observer sensitivity—is similar to anger, but a more complex, secondary emotion. Perpetrator sensitivity is associated with guilt, a secondary emotion as well (Schmitt et al., 2005, 2010; Lawrence, 2006). In adults, the core emotion underlying rejection sensitivity is fear, a withdrawal-oriented, primary emotion. In children and adolescents, anxious and angry rejection sensitivity is separately considered (Downey et al., 1998). The correlation of rejection sensitivity and trait anger in the present study indicates that anger may be a part of adult rejection sensitivity as well. The null-correlation of moral disgust sensitivity with trait anger and its negative links with aggression indicate that the core emotion underlying moral disgust sensitivity is indeed disgust, not anger (Pond et al., 2011). The present results also indicate that the experience of disgust in the face of moral norm violations is a fairly strong emotional reaction to this kind of behavior and may, therefore, have particularly strong influences on behavior in terms of withdrawal.

#### Time Perspective

Questionnaires on justice, moral disgust, and provocation sensitivity ask participants for typical responses in given situations, focusing the present; the rejection sensitivity questionnaire captures expectancies about rejection, thus focusing the future. In doing so, the rejection sensitivity questionnaire follows an expectancy-value conception which strives to explain behavior by multiplying the perceived value of an outcome with its probability of occurrence. Given that rejection sensitivity tended to show comparably low correlations with the other sensitivity measures and low loadings on a general interpersonal sensitivity factor in CFA, this conception in particular may distinguish rejection sensitivity from the other sensitivity measures in our study conceptually as well as statistically.

#### Perception of Others’ Behavior

All sensitivity measures base on the negative appraisal of behavior, but the reasons for this judgment differ. Justice sensitivity predisposes to perceiving behavior as unfair and morally wrong. Disgust sensitivity predisposes to perceiving others’ behavior as morally wrong, rejection sensitivity as hurting, and provocation sensitivity as hostile and provoking. In line with previous research, the perception of hostility and provocation in others seems to promote aggressive behavior in particular (Crick and Dodge, 1996; Lawrence and Hodgkins, 2009), whereas perceiving others’ behavior as morally wrong and hurting even to one’s own disadvantage may apparently result in both, aggression and withdrawal.

#### Correlations with Behavior

Relations with aggression differed between sensitivity measures (see below for details).

Based on these aspects, all sensitivity measures can be distinguished from one another. However, similarities are apparent, particularly between victim and provocation sensitivity. Victim sensitivity as opposed to provocation sensitivity, however, focuses on unjust cues in particular, does not require injustice to occur intentionally, considers cognitive reactions, and comprises a moral aspect as well.

### Relations of the Sensitivity Measures with Hostile Attributions and Trait Anger

Small, but significant positive correlations of victim sensitivity with hostile attributions are in line with previous research relating victim sensitivity to hostility (Schmitt et al., 2010). This backs the assumption that some of its negative effects might be explained by the tendency to attribute hostile intent to others (Gollwitzer et al., 2013; Bondü and Krahé, 2015), presumably by intensifying perceptions of injustice (Bondü and Richter, 2015). Correlations, however, were only small, indicating that hostile attributions can only account for parts of these negative effects. Positive correlations of provocation sensitivity with hostile attributions are not surprising, given that these attributions should be a prerequisite to perceive others’ behavior as provoking. Our findings, however, contradict earlier studies that did not find differences in hostility between individuals high and low in provocation sensitivity (Lawrence, 2006). Positive associations of moral disgust and hostile attributions may explain previous findings relating general disgust to the tendency to exclude persons who have violated moral norms (Faulkner et al., 2004). In contrast to our expectations, rejection sensitivity did not relate to hostile attributions. This may be due to the fact that the Adult Rejection Sensitivity Questionnaire (Berenson et al., 2009) merely captures anxious (and not angry) rejection sensitivity which does not necessarily require attributions of negative intent.

In line with theoretical assumptions and our expectations, victim and provocation sensitivity were positively correlated with trait anger (Schmitt et al., 1995). Again, these results contradict previous research that did not find differences in trait anger between individuals high and low in provocation sensitivity (Lawrence, 2006). In line with our assumptions, moral disgust did not correlate with trait anger, backing research suggesting that the core emotion underlying moral disgust is indeed disgust, not anger (Pond et al., 2011). Although the core emotion underlying rejection sensitivity in adults is considered fear or concern (Downey and Feldman, 1996), expecting rejection apparently may result in anger among adults as well. Thus, a mixture of fear and anger may explain the diverging relations of rejection sensitivity with different aggression measures. Relation patterns of the sensitivity measures with hostile attributions and trait anger were similar across genders, but only in men, observer sensitivity showed positive correlations with trait anger. This might explain why in men, observer sensitivity showed positive correlations with aggression.

### Interplay of the Sensitivity Measures, Hostile Attributions, and Trait Anger in Predicting Aggression

When only the sensitivity measures were considered as predictors for aggression, observer and particularly provocation sensitivity were consistent positive and perpetrator and moral disgust sensitivity consistent negative predictors of forms of aggression. Thus, experiencing disgust in the face of others’ norm violations may also protect from violating social norms oneself and to be more powerful than experiencing guilt after behaving immorally (as in perpetrator sensitivity). Even when controlled for hostile attributions and trait anger, physical aggression was better predicted by observer and provocation sensitivity that comprise aspects of anger and the tendency to retaliate. When hostile attributions and trait anger were controlled, victim sensitivity negatively predicted physical and verbal aggression and negative effects of rejection sensitivity on verbal aggression increased. This indicates suppressor effects, most likely due to the control of variance that anger accounts for, strengthening the impact of moral concerns associated with victim sensitivity and of fear associated with rejection sensitivity that should both counteract aggression.

Regarding functions of aggression, mostly in line with our assumptions, victim, rejection, and provocation sensitivity positively predicted reactive aggression. Also in line with our assumptions, negative effects of perpetrator and moral disgust sensitivity were particularly pronounced for proactive aggression, indicating that they protect from using aggression in order to reach egoistic goals in particular. Interestingly, they did not protect from reactive, that is, provoked aggression in the present study (for contradicting results: Pond et al., 2011; Bondü and Krahé, 2015; Bondü and Richter, 2015). In women, victim sensitivity predicted proactive aggression as well, indicating that victim sensitivity may not only lead to aggression in response to previous negative cues, but also to aggression that appears unprovoked and is used to reach egoistic goals (cf. Bondü and Krahé, 2015; Bondü and Richter, 2015). Hostile attributions and trait anger positively predicted proactive aggression, particularly in men, whereas trait anger—somewhat surprisingly and contradicting previous research (Crane and Testa, 2014)—did not relate to reactive aggression.

To sum up, the sensitivity measures added to the prediction of aggression, even when controlling for more global traits such as hostile attributions and trait anger. Moral disgust and perpetrator sensitivity were consistent negative predictors for forms of aggression in particular, whereas provocation sensitivity was a consistent positive predictor, supporting the assumption that provocation is a primary cause for aggression (Anderson and Bushman, 2001). Thus, in line with the cognitive–affective system theory of personality (Mischel and Shoda, 1995), the sensitivity measures apparently influence the encoding of, interpretation of, and response to certain situations and may account for some of the behavioral variability in given situations.

### Limitations and Outlook

The strengths of this study include the use of multiple interpersonal sensitivities and aggression measures and the consideration of hostile attributions and trait anger. Limitations include the marginally acceptable internal consistencies of rejection sensitivity and verbal aggression, differences in sample sizes between men and women, and the lack of control for broader personality traits such as neuroticism. Finally, our study is cross-sectional and does not allow for causal inferences.

Future research should, therefore, replicate the present findings with longitudinal data. It may want to consider interaction effects of the sensitivity measures. Given the mixed effects of rejection sensitivity on aggression and its association with anger, future research should distinguish anxious and angry rejection sensitivity in adults as well. Other sensitivity measures (e.g., frustration sensitivity; Lawrence, 2006) may be considered. Hostile attributions and trait anger are eligible to control for negative feelings and attitudes associated with aggression, but empathy and perspective taking may be superior control variables for moral concerns underlying observer, perpetrator, and moral disgust sensitivity (Schmitt et al., 2005). Finally, future research should examine the processes explaining gender differences in the links of the sensitivity measures and aggression.

Despite their cross-sectional nature, the results of the present study show that the sensitivity measures cannot be integrated to one single factor of interpersonal sensitivity and that they add to the prediction of aggression. This highlights the importance of considering contextual interpersonal differences measures in researching aggression and in planning adequate prevention measures. This is important, because these dispositions affect the perception and interpretation of social cues in the long run and, therefore, are important to address in order to prevent a self-perpetuating circle of negative social cues and increasing sensitivities toward these cues. Importantly, our results indicate that these effects cannot be better explained by other well-known risk factors for aggression and broader personality traits such as hostile attributions or trait anger.

## Author Contributions

Both authors have made substantial contributions to the conception of the work and the acquisition, analysis, or interpretation of data for the work; have contributed to drafting and revising the manuscript; gave their approval of the version to be published, and agreed to be accountable for all aspects of the work in ensuring that questions related to the accuracy or integrity of any part of the work are appropriately investigated and resolved.

## Conflict of Interest Statement

The authors declare that the research was conducted in the absence of any commercial or financial relationships that could be construed as a potential conflict of interest.
